# Pharmacoeconomic Analysis of the Different Therapeutic Approaches in Control of Bovine Mastitis: Phytotherapy and Antimicrobial Treatment

**DOI:** 10.3390/antibiotics12010011

**Published:** 2022-12-22

**Authors:** Zorana Kovačević, Jovan Mihajlović, Snežana Mugoša, Olga Horvat, Dragana Tomanić, Nebojša Kladar, Marko Samardžija

**Affiliations:** 1 Department of Veterinary Medicine, Faculty of Agriculture, University of Novi Sad, Trg Dositeja Obradovica 8, 21000 Novi Sad, Serbia; 2 Mihajlović Health Analytics (MiHA), Omladinskih radnih akcija 54, 21000 Novi Sad, Serbia; 3 University Medical Center Groningen, 9713 GZ Groningen, The Netherlands; 4 Faculty of Medicine, University of Novi Sad, Hajduk Veljkova 3, 21000 Novi Sad, Serbia; 5 Faculty of Medicine, University of Montenegro, Krusevac bb, 81000 Podgorica, Montenegro; 6 Institute for Medicine and Medical Devices of Montenegro, Bulevar Ivana Crnojevića 64a, 81000 Podgorica, Montenegro; 7 Department of Pharmacology and Toxicology, Faculty of Medicine, University of Novi Sad, Hajduk Veljkova 3, 21000 Novi Sad, Serbia; 8 Center for Medical and Pharmaceutical Investigations and Quality Control, Department of Pharmacy, Faculty of Medicine, University of Novi Sad, Hajduk Veljkova 3, 21000 Novi Sad, Serbia; 9 Clinic for Reproduction and Obstetrics, Veterinary Faculty, University of Zagreb, Heinzelova 55, 10000 Zagreb, Croatia

**Keywords:** dairy cows, essential oils, Phyto-Bomat, antimicrobials, milk yield, costs, effectiveness

## Abstract

Mastitis in dairy cows is responsible for major economic losses on dairy farms worldwide as the most expensive and prevalent disease in dairy cattle. In spite of the fact that antibiotic therapy still remains the main treatment strategy for bovine mastitis, concerns about the shortcomings of this treatment approach are continuously raised. Hence, research on alternative treatments with increased effectiveness and reduced costs is needed. Therefore, we conducted a pharmacoeconomic analysis of conventional antibiotic vs. a proposed Phyto-Bomat treatment based on essential oils in bovine mastitis therapy. Treatments were compared from the farmer’s perspective in the domain of costs (expressed in total, direct and indirect, cost differences) and effectiveness (expressed in daily milk yield differences). Economic calculations were based on data from a dairy farm in Serbia. The average cost of conventional antibiotic treatment was estimated at EUR 80.32 consisting of therapy costs, veterinary services and milk rejection costs at EUR 16.54, EUR 17.68 and EUR 46.10, respectively. The average cost of Phyto-Bomat treatment was estimated at EUR 76.34 with therapy costs of EUR 34.34, veterinary costs of EUR 32.00 and rejection of milk costs of only EUR 10.00. Therefore, Phyto-Bomat results in cost savings approximating EUR 4 per each mastitis episode with the highest cost reductions obtained in milk rejection costs. This estimation of Phyto-Bomat’s economic benefits could be used as a starting point for the inclusion of this formulation as an alternative treatment approach with a focus on subclinical mastitis since it contributes to most of the financial losses.

## 1. Introduction

Bovine mastitis is a disease of major economic importance in the dairy industry worldwide since the prevention and treatment of this disease represent a significant financial burden for milk producers due to large milk production losses, increased veterinary costs, increased cow mortality and negative effects on animal welfare, and also the impact on human health and food safety [1,2]. Moreover, the economic losses associated with mastitis are due to increased production costs, the negative impact on milk production, therapy costs and other costs related to the cow with mastitis [3]. Therefore, mastitis is the most economically significant disease affecting the dairy industry, with costs of over EUR 124 (=USD 147) per cow per year, resulting in losses of EUR 500 million, EUR 3 billion and EUR 125 billion in Germany, the EU and worldwide, respectively [4,5]. Furthermore, the total loss of milk due to clinical mastitis in dairy cows is estimated to be between 110 and 550 L of milk per lactation, depending on the individual cow [6].

When it comes to the public health issue, the increase in incidence of mastitis in dairy cows generally results in the increased use of antimicrobials, resulting in an augmented content of antibiotic residues in milk and the potential for resistance to antimicrobials resulting in an increase in health care costs [2]. Therefore, in order to limit the use of antibiotics to reduce the risks of antibiotic residues in the milk, researchers all over the world are trying to find different alternatives to antibiotic therapy, such as the use of homeopathy, concerning efficacy and safety, both for animals and for consumers [7,8], as well as the dietary zeolite clinoptilolite [9]. Moreover, phytotherapy has become more and more popular, especially the use of essential oils (EOs)-based pharmaceutical formulation in mastitis treatment [10,11].

Bovine mastitis is known as a mammary gland inflammation, which may be considered in a clinical or subclinical form of the disease [12]. It is difficult to estimate the losses associated with clinical mastitis, and even more difficult to quantify those associated with the subclinical form [13]. Actually, clinical mastitis is characterized by visible abnormalities and clear clinical signs, such as an edema, an increase in temperature, pain in the mammary gland or any change in milk composition and appearance [1,14]. In addition, subclinical disease does not manifest in visible changes, and can only be diagnosed by laboratory tools through an increased milk somatic cell count, and therefore, it is not easily recognized by farmers [5,15]. Furthermore, the incidence of subclinical mastitis is estimated to be 15–40 times higher than for clinical. Hence, due to its higher frequency and capacity to reduce milk yields while going unnoticed, subclinical mastitis has the potential to result in an even higher economic burden [5,16]. For example, losses due to subclinical mastitis are estimated to outreach the economic losses of clinical mastitis by 3–4 times [14,17]. Additionally, milk production decreases by 17.2% in subclinical mastitis without the occurrence of any obvious clinical signs [14].

In general, mastitis-caused economic losses can be divided into two categories, direct and indirect costs [18,19]. Direct costs are also known as hidden costs and they include veterinary services, diagnostics, treatment, additional labor and discarded milk (during treatment), while indirect costs are defined as those that are not always obvious to the milk producer. Furthermore, indirect losses due to subclinical mastitis are not well recognized by many farmers, but they include reduced milk yield, premature rejection losses and reduced quality premiums [16,20]. While subclinical mastitis has no direct costs, it has been shown to be responsible for most of the economic losses due to mastitis, with a reduction in milk production being the main factor [21]. However, the economic impact of mastitis varies and should be calculated at the farm or herd level, and it depends on local, regional, epidemiological, managerial and economic conditions [16].

Pharmacoeconomics is a field of economics that compares the costs and outcomes of various pharmaceutical products and treatment strategies [22]. There are established guidelines for pharmacoeconomic evaluation [23] as well as current efforts and proposals to reduce healthcare costs in Serbia [24]. In addition, pharmacoeconomic assessments in human medicine in Serbia have been published for targeted cancer therapies [25,26,27]. According to the authors’ knowledge, there have been no publications relating to the pharmacoeconomics of veterinary medicines to date.

Therefore, the aim of our study was to conduct pharmacoeconomic analysis in veterinary medicine and to analyze the differences in the cost and effectiveness of conventional antibiotics vs. EOs treatment in bovine mastitis therapy in Serbia.

## 2. Results

### 2.1. Milk Yield Data in the Standard Lactation

Information necessary for pharmacoeconomic analysis are data for the total cow population (real-world evidence). The average milk yield for 100 cows in standard lactation (305 days) and the daily milk yield of those cows are presented in Table 1. The average milk yield of cows with diagnosed clinical mastitis in one lactation (8360 L) is less than in those cows without mastitis (9200.71 L). Moreover, the daily yield of milk during the period of lactation in cows with diagnosed mastitis is less than in those without diagnosed clinical mastitis, 28.4 L and 29.89 L, respectively.

### 2.2. Determination of Antibiotic Costs in the Treatment of Mastitis

The antibiotics used in mastitis therapy in the case of the total cow population are presented in Table 2. For each of them, the recommended duration of therapy is given with respect to the Summary of the Product’s Characteristics (SmPC) of each drug. Based on the antibiotic’s price and the dose used in the treatment of mastitis, the price per dose of the drug was calculated. Furthermore, based on the recommended duration of the therapy, the cost of antibiotic therapy per episode of mastitis was calculated, which is significant for pharmacoeconomic analysis. In addition, the withdrawal periods for the antibiotics used in the treatment of mastitis, based on the costs of rejected milk, were determined (Table 2).

### 2.3. Determination of the Total Average Cost of Mastitis

The calculations of the costs significant for the calculation of the average cost of mastitis in the analyzed sample are presented in Table 3. These costs are based on the sample of 100 cows representing the data for the total cow population (real-world evidence).

### 2.4. Determination of Phyto-Bomat Cost in the Treatment of Mastitis

The calculations of the costs in the case of phytotherapy (treatment with Phyto-Bomat) are presented in Table 4 and Table 5. Actually, the price of Phyto-Bomat in mastitis therapy is calculated for one cow based on the price of oil and their volume needed to make the pharmaceutical formulation, as well as based on the price of the injectors (Table 4). The price calculation was made on the duration of therapy (5 days) and dosage interval (twice a day). Actually, 10 injectors are needed for the treatment of one cow. Thus, the cost per cow is EUR 34.34 ± 3.43 (Table 4).

Furthermore, the total cost of therapy with Phyto-Bomat in the case of mastitis is presented in Table 5.

### 2.5. Determination of the Cost Differences between Antimicrobial and Phyto-Bomat Treatment

The cost difference between conventional treatment with antibiotics and phytotherapy is presented in Table 6. Furthermore, based on the costs calculated for each episode of mastitis, Phyto-Bomat has the potential to save EUR 4.00.

### 2.6. Daily Milk Yield Determination before and after Antibiotic and Phyto-Bomat Treatment

**Figure 1:**
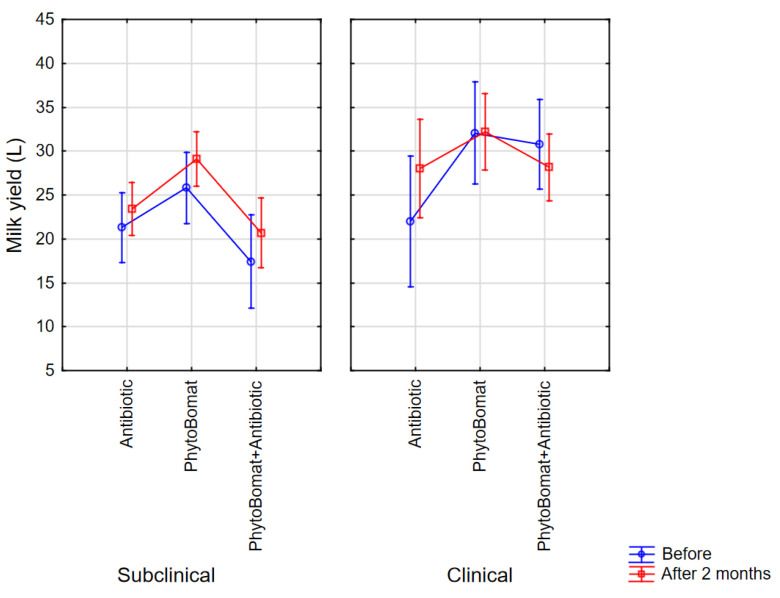
Daily milk yield before and 2 months after the completion of different treatments (antibiotics, Phyto-Bomat and Phyto-Bomat + antibiotic). Vertical bars denote 0.95 confidence interval.

The application of Factorial ANOVA on data describing the milk yield (L) in cows with clinical or subclinical mastitis shows that each of the evaluated factors, the form of the applied treatment (F(4, 150) = 3.78, Wilks λ = 0.82, *p* = 0.006) and the form of the mastitis (F(2, 75) = 7.12, Wilks λ = 0.84, *p* = 0.001) significantly affect the milk yield (F(6, 148) = 2.40, Wilks λ = 0.83, *p* = 0.03), but the combined effect of these factors is not of statistical significance (F(4, 150) = 1.44, Wilks λ = 0.83, *p* = 0.22) (Figure 1).

The post hoc Fisher LSD test indicates that the form of mastitis is of interest for milk yield only in the case of treatment with the combination (Phyto-Bomat and antibiotic) (*p* = 0.001). Furthermore, the treatment choice affected the milk yield in the case of clinical mastitis treated with Phyto-Bomat, as well as with antibiotics (*p* = 0.037).

## 3. Discussion

To the best of our knowledge, this is the first study where pharmacoeconomic analysis was applied in the field of veterinary medicine. The reason why this was the first attempt could be difficulties in data collecting regarding the specialties presented in the veterinary field. Furthermore, in most economic evaluations in human medicine, the future monetary costs and benefits and future health benefits are discounted at the same rate [41], whereas cost–benefit analysis measures outcomes in dollars, while cost-effectiveness analysis measures outcomes in nonmonetary units [42]. These principles should also be applied to veterinary medicine, although is not easy to obtain the data on nonmonetary units, i.e., health benefits, such as the mortality rate, prolonged life/lactation period and improvements in the quality of life. Actually, it is well known that in human medicine a well-designed pharmacoeconomic analysis involves 10 steps, such as defining the problem, determining the study’s perspective, determining the alternatives and outcomes, selecting the appropriate pharmacoeconomic method, placing monetary values on the outcomes, identifying study resources, establishing the probabilities of the outcomes, applying decision analysis, discounting costs or performing a sensitivity or incremental cost analysis, and presenting the results, along with any limitations of the study [42]. Bearing this in mind, in our study, all steps mentioned above were applied in order to undertake a pharmacoeconomic evaluation with the greatest likelihood of obtaining valid and useful results.

Direct costs are more obvious and can be easily calculated. Indirect costs are more difficult to calculate and are sometimes the result of various factors that affect the animal itself. Thus, in addition to mastitis, other health disorders can lead to premature rejection and the cost of replacing the animal [43]. From the point of view of dairy farmers, mastitis decreases the earning potential of affected cows as well as the profitability of the entire dairy business [44]. Furthermore, the only nonmonetary units used in our study were the withdrawal period and milk yield in the standard lactation after both applied treatments, conventional antimicrobial treatment vs. phytotherapy with the proposed EOs-based pharmaceutical formulation (Phyto-Bomat). These two parameters are considered as the most important costs in mastitis treatment.

Economic losses due to clinical and subclinical mastitis vary significantly among countries, depending on factors such as milk price, and treatment and veterinary service costs [45]. Reduced milk production is the major cost associated with subclinical mastitis and a substantial cost associated with clinical mastitis [46]. The estimated milk yield reduction caused by mastitis varies across studies because it can be influenced by the age and breed of the cow, morphological characteristics of the udder, stage of lactation, milk yield before mastitis occurred, mastitis-causing organisms, inflammation grade, diagnosis (early or late after occurrence), type of treatment, feeding practices, etc. [45,47]. Because of these difficulties, study results may not always be directly comparable [48]. Despite country-specific variation, long-term milk yield losses constitute a notable share of the economic losses attributable to mastitis [45]. The difference between 305 d milk yields and the daily milk yield in our study with and without mastitis shows how intramammary infection decreases milk production, which is in accordance with the literature data [46,49]. The reduction in milk production is largely due to physical damage to the mammary parenchyma of the affected mammary gland [50]. However, it must be stated that the inflammation can cause decreased appetite and lower food intake due to pain and decreased movement, which leads to a decrease in milk production [45].

When clinical mastitis occurs, additional costs result from discarded milk, and the use of drugs and veterinary services. Antibiotics are commonly used to treat clinical mastitis episodes [46]. Residues of antibiotics are excreted in the milk of treated cows for different lengths of time and in different concentrations, depending, above all, on the type of antibiotic. Such milk is discarded so that it does not enter the food chain. Furthermore, the administration of antibiotics usually requires a withdrawal time, during which milk must be discarded or fed to calves [51]. This cost can be substantial, which is confirmed by our study, where the cost for rejecting milk due to the withdrawal period was bigger than the cost of antibiotics for treating. When it comes to the withdrawal period regarding the Phyto-Bomat treatment, there is an advantage since in our previous work it was calculated to be 24 h [52]. In addition, based on our research results, the withdrawal period for antibiotics used in mastitis treatment is 3.5 days on average and could be even higher (up to 7) (Table 2). This makes the cost of the rejected milk higher in the case of the treatment with antibiotics. Since some researchers have suggested that rejected milk is one of the most expensive costs associated with mastitis [46,53], this makes our research results valuable in the calculation of the cost of treatment with phytotherapy.

Our research results show that considering the milk yield, each of the evaluated factors, such as the form of the applied treatment and the form of the mastitis, significantly affect the milk yield, but the combined effect of these factors is not of statistical significance (Figure 1). The form of mastitis is of interest for the yield of milk only in the case of treatment with the combination (Phyto-Bomat and antibiotic) (*p* = 0.001). Furthermore, the treatment choice affects the milk yield only in the case of clinical mastitis treated with Phyto-Bomat, as well as with antibiotics (*p* = 0.037), which is in agreement with other results where an increase in milk yield after subclinical mastitis treatment with herbal spray and Mastilep^®^ gel was reported [54]. Furthermore, an increase in milk production was recorded in a group of cows treated with Mastilep^®^ only [55]. Contrary to our findings, Mullen et al. [56] detected no significant differences among treatments in terms of milk yield differences between the lactation following treatment and the lactation preceding treatment.

The benefits of the treatment of cows with mastitis are easy to calculate. If the number of cows in the Republic of Serbia is ~200,000 (estimates about the steady decline in the number of cows from 1975–2009 indicate that in 2009 it was ~500,000 [57]), and the prevalence of clinical mastitis is 15% [58], possible savings in Serbia by replacing antibiotics with Phyto-Bomat could be ~ EUR 117,432 per year.

## 4. Materials and Methods

### 4.1. Determination of the Average Milk Yield in the Standard Lactation

The data necessary for pharmacoeconomic analysis were taken from a computer database kept by veterinarians employed on a dairy farm located in the Vojvodina district, Serbia. These data were used as the real-world evidence data (for the total cow population) in this study. Actually, they were collected on a farm with 1140 cows during a period of one year. Only cows with milk yield data available for each lactation were included in the study to calculate daily milk yield and milk yield in the standard lactation. Moreover, cows with lactation in progress were excluded, so the data for 100 cows were taken into account. Hence, the daily milk yield and milk yield in the standard lactation were calculated for cows with and without diagnosed mastitis.

### 4.2. Determination of the Antibiotic Cost in the Treatment of Mastitis

As a real-world evidence data for the pharmacoeconomic analysis of antibiotic treatment, the following data were collected: cost of antimicrobials per episode of mastitis, cost of veterinary services per episode of mastitis and cost of milk rejection due to withdrawal period. Furthermore, the price list of antimicrobials was obtained from the local veterinary pharmacy, while the price of veterinary services was obtained from the price list of veterinary services officially proposed and approved by the Veterinary Chamber of Serbia [59]. Regarding the veterinary services provided during mastitis treatment, the key opinion leaders from this field in the Republic of Serbia were consulted. In addition, the average duration of mastitis was also calculated and taken into consideration.

Based on data from a computer database, antibiotics used in the treatment of the mastitis were collected. The selected antibiotics were then divided based on the route of administration, duration of therapy, dose and withdrawal period. Based on the prices of the package of each antibiotic used in mastitis treatment obtained from local veterinary pharmacies, the dose and the length of therapy, the cost of each of these antibiotics was calculated per episode of mastitis, all in accordance with the Summary of the Product’s Characteristics. In addition, the cost of mastitis therapy was calculated in relation to the antibiotic therapy used in the analyzed sample. This value is expressed as the average value of the analyzed sample.

### 4.3. Determination of the Total Cost of Average Mastitis

The total cost of average mastitis was calculated taking into consideration the following: number of cows analyzed (cows from the real-world evidence data for 100 cows), the average duration of mastitis, the cost of the drug (antibiotic) per episode of mastitis, the cost of veterinary services, as well as the average cost of rejecting milk per episode of mastitis.

### 4.4. Determination of Phyto-Bomat Cost in the Treatment of Mastitis

The cost of Phyto-Bomat treatment in mastitis in cows was also calculated. Phyto-Bomat is intramammary pharmaceutical formulation, based on four EOs, such as common (*Thymus vulgaris* L.) and wild thyme (*Thymus serpyllum* L.), oregano (*Origanum vulgare* L.) and mountain savory (*Satureja montana* L.), diluted with common marigold (*Calendula officinalis* L.) and St. John’s wort (*Hypericum perforatum* L.) oil macerates (herbal drug–sunflower oil, 1:5) in an amount of up to 15 mL in an intramammary injector, as described by Kovačević et al. [52], while chemical composition and antimicrobial activity of selected EOs was described by Kovačević et al. [60,61]. The analysis was performed according to the defined therapeutic protocol for the treatment of mastitis in one cow (two times per day, for 5 days), while the price of phytotherapy treatment was calculated by taking into account the price of the applied pharmaceutical formulation (cost of ingredients and the packaging), as well as the cost of the veterinary services and the cost of milk rejection, representing the total cost of Phyto-Bomat therapy per cow. Furthermore, in order to determine the cost of Phyto-Bomat therapy, the prices of EOs and base oils included in the formulation were taken into account, as well as the price of injectors used for application of the proposed formulation. Based on the price of the given oils, as well as their volumes required for making the intramammary pharmaceutical formulation, the price of the Phyto-Bomat was calculated.

### 4.5. Determining the Cost of Veterinary Services in Mastitis

Veterinary services designated for this analysis were: the intramammary application of the drug, application of the drug parenterally (intramuscularly and subcutaneously), mastitis rapid test (diagnosis) and udder control and sampling. The prices of veterinary services used in the treatment of mastitis were taken from the recommended price list of veterinary services proposed by the Veterinary Chamber of Serbia. The cost of veterinary services in the treatment of mastitis were expressed by the episode of mastitis.

### 4.6. Cost of Milk Rejection

The withdrawal period of the given antibiotic, impacts on how long the rejection of milk will last, which also affects the cost-effectiveness of antibiotic therapy. The price of milk rejection was calculated by multiplying the withdrawal period of a given antibiotic with the price of daily milk yield for each cow. The calculation of costs should also take into account the withdrawal period for the proposed EOs-based pharmaceutical formulation (Phyto-Bomat). The amount of dominant components (thymol and carvacrol) in Phyto-Bomat were determined in milk of treated cows in our previous work [52]. Actually, within period of 24 h, for both compounds, the milk samples were at the same levels as before treatment.

### 4.7. Determination of the Cost Differences between Antimicrobial and Phyto-Bomat Treatment

The cost differences between antimicrobial and Phyto-Bomat treatment were calculated taking into account following costs: the therapy, the veterinary services and the rejection of the milk.

### 4.8. Determination of Indirect Costs Accrued by the Loss in Daily Milk Yield

Everyday data on milk production of cows were entered into the database by veterinarians after milking. In order to calculate and compare the daily yield of cows with diagnosed mastitis, the data after antibiotic therapy were taken, as well as after Phyto-Bomat therapy. Additionally, we took data of milk yield after combined therapy (antibiotic + Phyto-Bomat). The obtained values with the data of daily milk yield before all type of treatments were compared in the case of subclinical and clinical forms of mastitis.

### 4.9. Data Analysis

The obtained results were processed and summarized by the program Microsoft Office Excel (v2019). The data were expressed as mean values. Furthermore, the obtained data were statistically processed by Tibco Statistica v13.5.

All of the variables were characterized by application of descriptive statistics. Factorial ANOVA was applied for the assessment of clinical form of mastitis and applied treatment influence on milk yield. The results were considered significant if *p* < 0.05.

## 5. Conclusions

This study represents the implementation of pharmacoeconomic analysis for the first time in the veterinary medicine of Serbia in order to assess the clinical and economic value of the alternative, herbal-based mastitis treatment, Phyto-Bomat, and its potential to enhance the health of humans and animals. Furthermore, the analysed pharmaceutical formulation can help decrease AMR, which appears to be one of the major public health challenges. The significant results of this study indicate economic benefits (savings) for farmers associated with the use of the Phyto-Bomat treatment. Finally, the use of Phyto-Bomat in subclinical mastitis can potentially decrease the use of antimicrobials by preventing the development and consequential antibiotic treatment of clinical mastitis.

## Figures and Tables

**Table 1:** Daily milk yield and milk yield in the standard lactation (milk yield = 305 days in lactation) in cows with and without diagnosed clinical mastitis from the total cow population (average ± SD).

Milk Yield in the Standard Lactation (L)		Daily Milk Yield (L)	
with Mastitis	without Mastitis	with Mastitis	without Mastitis
8360 ± 1251	9200.71 ± 1552.03	28.54 ± 4.41	29.89 ± 4.69

**Table 2:** The antibiotics used in mastitis therapy.

Brand Name	INN *	Route of Application	Withdrawal Period	The Price of the Package (EUR)	Price Per Dose of Drug (EUR)	Cost Per Episode of Mastitis (EUR)
**Mastijet^®^** **[28]**	tetracycline, neomycin, bacitracin, prednisolone	intramammary	5	60	8.5	34.2 ± 3.4
**Tilozin 200^®^** **[29]**	tylosin	parenteral	7	5.8	1.7	5.4 ± 0.5
**Penstrep^®^** **[30]**	procaine benzylpenicillin, dihydrostreptomycin	parenteral	2.5	8	2.5	6.4 ± 0.6
**Cefimam^®^** **[31]**	cefimam	intramammary	5	2.5	2.5	7.5 ± 0.7
**Kelbomar^®^** **[32]**	marbofloxacin	parenteral	3	51	6	18.3 ± 1.8
**Tetra delta^®^** **[33]**	novobiocin, neomycin, procaine benzylpenicillin, dihydrostreptomycin, prednisolone	intramammary	4.5	3	3	3 ± 0.3
**Medilozin^®^** **[34]**	tylosin	parenteral	4.5	5	1.3	5.4 ± 0.5
**Neoceftiofu^®^ HCl 5%** **[35]**	ceftiofur hydrochloride	parenteral	0	7.7	1	3 ± 0.3
**Veyx yl LA 200^®^** **[36]**	amoxicillin	parenteral	3	13	3.9	11.7 ± 1.2
**Rilexine 200 LC^®^** **[37]**	cefalexin	intramammary	3	30.5	2.5	10.2 ± 1.0
**Ceftionel 50^®^** **[38]**	ceftiofur	parenteral	0	26.3	3.41	10.2 ± 1.0
**Enrocin-S 10%^®^** **[39]**	enrofloxacin	parenteral	4	5.5	0.8	3.3 ± 0.3
**Synulox^®^** **[40]**	amoxicillin/ clavulanic acid	intramammary	3.5	3.2	3.2	9.5 ± 0.9

* INN, International Nonproprietary Name.

**Table 3:** Total cost of mastitis in the analyzed sample of 100 cows expressed in euros (EUR) (average ± SD).

Average duration of mastitis (days)	3.5 ± 1.0
The average cost of the drug (antibiotic) per episode of mastitis	16.54 ± 8.83
The cost of veterinary services	17.68 ± 4.62
The average cost of rejecting milk per episode of mastitis	46.10 ± 24.59
Total cost	80.32

**Table 4:** The total cost of the Phyto-Bomat per cow expressed in euros (EUR).

EOs		
	*Thymus vulgaris* L.	2.64 ± 0.26
	*Thymus serpyllum* L.	5.30 ± 0.53
	*Origanum vulgare* L.	5.30 ± 0.53
	*Satureja montana* L.	4.60 ± 0.46
Drug vehicle		
	*Calendula officinalis* L.	0.78 ± 0.07
	*Hypericum perforatum* L.	0.72 ± 0.07
Injectors		15.00 ± 1.5
Total		34.34 ± 3.43
Price per injector		3.43 ± 0.34

**Table 5:** Total cost of Phyto-Bomat therapy per cow per episode of mastitis expressed in euros (EUR).

The cost of the Phyto-Bomat	34.34 ± 3.43
The cost of veterinary services	32.00 ± 3.20
The cost of milk rejection	10.00 ± 1.00
Total cost	76.34 ± 7.63

**Table 6:** The cost difference between antibiotic treatment and phytotherapy in euros (EUR).

The Type of the Cost	Antibiotic Treatment (EUR)	Phyto-Bomat Treatment (EUR)
The therapy	16.54	34.34
The veterinary services	17.68	32.00
The rejection of milk	46.10	10.00
Total costs	80.32	76.34
The difference in the total costs	4.00	

## Data Availability

The data used to support the findings of this study are available in the present manuscript.
